# Raman spectroscopic investigation of the interaction of *Enterococcus faecalis *and vancomycin: towards a culture-independent antibiotic susceptibility test

**DOI:** 10.1186/cc11772

**Published:** 2012-11-14

**Authors:** U Neugebauer, C Assmann, U Schröder, A Ramoji, U Glaser, C Beleites, W Pfister, J Popp, M Bauer

**Affiliations:** 1 Center for Sepsis Control and Care, Jena University Hospital, Jena, Germany; 2 Institute of Photonic Technology, Jena, Germany; 3 Institute of Medical Microbiology, Jena University Hospital, Jena, Germany; 4 Institute of Photonic Technology, Institute of Physical Chemistry and Abbe Center of Photonics, Jena, Germany

## Background

*Enterococcus faecalis *gained importance in the last years for causing tough and troublesome nosocomial infections especially in the urinary tract, sometimes even leading to sepsis. Causes of concern are increasing resistances of enterococci. Therefore, it is of utmost interest to identify resistance patterns of bacteria within a very short timeframe to select the right therapy and efficiently prevent spreading. Within our junior research group we want to develop an on-chip device to probe antibiotic susceptibility patterns of sepsis pathogens based on optical spectroscopy without the need for time-consuming bacterial cultivation. In this contribution, we present our first results with a focus on *E. faecalis*.

## Methods

*E. faecalis *was grown in liquid culture and characterized in the presence and absence of vancomycin by means of Raman spectroscopy. Multivariate statistical analysis was applied to extract the spectral differences due to antibiotic treatment. To enable the spectroscopic analysis directly with patient samples (such as urine) Raman spectroscopy was combined with dielectrophoresis. Bacteria from suspensions were captured and kept at well-defined positions in a nonuniform electric field for the time of Raman measurement. Currently, bacteria-spiked model urine is used. However, the investigations shall be extended to urine from patients.

## Results

**Figure 1:**
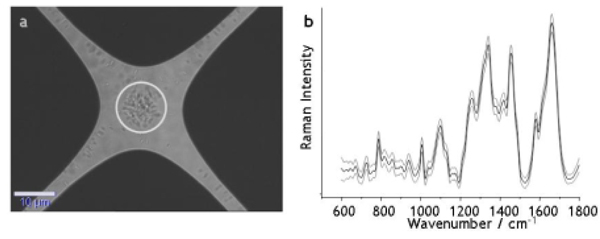
**(a) *E. faecalis *trapped between the electrodes on a quadrupole dielectrophoretic chip**. **(b) **Raman mean spectra with standard deviation taken from 1,000 spectra of the trapped *E. faecalis*.

A clear distinction between Raman spectra of *E. faecalis *with and without antibiotic treatment is possible even 30 minutes after incubation with vancomycin. A quadrupole electrode design is presented that allows the efficient capturing of *E. faecalis *and *Escherichia coli*, by means of negative dielectrophoresis [1] (Figure 1a). From the captured bacteria in solution on the dielectrophoretic chip, high-quality Raman spectra have been recorded within 1 second (Figure 1b). These spectra allowed a reliable differentiation of the two commonly encountered bacterial species in urinary tract infections: *E. faecalis *and *E. coli*. First steps have been undertaken to implement such dielectrophoretic capturing structures into a microfluidic device for simplified sample handling.

## Conclusion

Raman spectroscopy in combination with statistical analysis holds the potential for a fast evaluation of bacterial antibiotic susceptibility without the need of time-consuming cultivation.
